# Supplementary material for: The Association between 4-Tertiary-Octylphenol, Apoptotic Microparticles, and Carotid Intima-Media Thickness in a Young Taiwanese Population
Source: Toxics. 2023 Sep 6;11(9):757. doi: 10.3390/toxics11090757 (PMC10537624; doi:10.3390/toxics11090757)
Supplement: Supplementary file 1 [file toxics-11-00757-s001.zip › toxics-2571587-supplementary.pdf]

Supplementary Material

S1.1. Study population and data collection

From 1992 to 2000, approximately 2,615,000 to 2,932,000 school-age children in grades 1 to 12 received an annual urine screening of urine strip by the Chinese Foundation of Health in Taipei, Taiwan. Subjects with abnormal results from two tests for proteinuria, glycosuria, or hematuria underwent a third urine screening test and a general health check-up. A total of 103,756 school children received the health check-ups and the third urine screen. Among these children, 9,227 had elevated blood pressure and 94,529 had normal blood pressure.

From 2006 to 2008 we established a cohort, the YOung TAiwanese Cohort (YOTA) study, based on students with and without childhood elevated blood pressure, selected from the 1992–2000 urine mass screening population. In the follow-up, we mailed invitation letters to eligible students in the Taipei area. After 3–5 days, 12 trained assistants and nurses conducted telephone interviews inviting those subjects with childhood elevated blood pressure to come in for a follow-up health examination. No telephone interview contact was made with normotensive students. Among the 707 subjects with elevated blood pressure in childhood, 303 completed the follow-up health examinations, giving a response rate of 42.9%. Among the 6,390 subjects with normal BP in childhood, 486 completed the follow-up health examinations, giving a response

rate of 7.6%. In order to differentiate the effects of environment on age of exposure, we recruited 97 subjects as “best friend controls” in the cohort follow-up period. A total of 886 subjects were included in this study. Physical check-ups were given after written informed consent at National Taiwan University Hospital. All methods in this study were performed in accordance with the relevant guidelines and approved by the Research Ethics Committee of at the National Taiwan University Hospital. All the 886 subjects were enrolled in the present study.

S1.2. Measurement of CIMT

The detailed method has been introduced in our previous studies [1]. CIMT was defined as the distance from the front edge of the first echogenic line (i.e., lumen-intima interface, the interface between the lumen and vascular intima) to the front edge of the second echogenic line (i.e., media-adventitia interface, the interface between the vascular media and adventitia) in the far wall of the vessel. An experienced technician used a high-resolution B-mode ultrasonography (GE Vivid ultrasound system, Horten, Norway) equipped with a 3.5–10 MHz real-time B-mode scanner to examine the CIMT of extracranial carotid arteries; we then applied a software package for vascular ultrasound for off-line automatic calculations. The CIMTs of the common carotid artery (CCA) proximal to the carotid bifurcation, bulb,

and internal carotid artery (ICA) were obtained bilaterally. CCA1 and CCA2 are specific point located 0–1 cm and 1–2 cm, respectively, along the CCA distal to the carotid bifurcation. We averaged CCA1 and CCA2 to obtain a mean that is representative of the CCA. Mean CIMT in this analysis was determined by averaging four measurements on bilateral CCAs. To determine the reliability of repeated measurements, the technician conducted a second reading on 30 randomly selected subjects 2 weeks later. The reliability of the CIMT measurement from bilateral CCAs (i.e., the mean of the right and left CCA) had excellent intra-observer coefficient of correlation reliability (ICCR), approximately 98.8% and 98.5%, respectively.

S1.3. Covariates

Sociodemographic data were recorded during the study. In terms of lifestyle habits, alcohol consumption was divided into two groups -0 "current drinking" and "currently not drinking" -- and smoking status was classified as "active smoker" or "inactive smoker". Household income was classified as "greater than Taiwan dollars (TWD) 50,000 per month" or "less than TWD 50,000". For participants ≥ 20 years old, body mass index (BMI) z scores were calculated using the formula (BMI for each participant - mean of BMI)/(standard deviation of BMI), while for subjects 12-19 years old, BMI was calculated using the WHO anthropometric calculator [2].

Serum triglyceride (TG), high-density lipoprotein cholesterol (HDL-C), low-density lipoprotein cholesterol (LDL-C), and uric acid concentrations were measured by an automatic analyzer (Technician RA 2000 Autoanalyzer, Bayer Diagnostic, Mishawaka, IN, USA). The homeostasis model assessment of insulin resistance (HOMA-IR) index is considered a good indicator of insulin resistance in adolescents and adults [3]. Diabetes was defined as fasting blood glucose level ≥126 mg/dL or current use of oral hypoglycemic agents or insulin. In adults, hypertension was defined as a mean blood pressure ≥140/90 mm Hg or current use of antihypertensive medications. Adolescent hypertension was determined by repeated-measures blood pressure values as systolic blood pressure (SBP) or diastolic blood pressure ≥ the age-, sex- and height-specific 95th percentiles [4]

Supplementary Table S1. Linear regression coefficients (95% CI) of apoptotic microparticles and CIMT with a unit increase in ln 4-t-OP concentrations in the total population and subpopulations of the subjects.

		Ln CD31+/CD42a− (counts/μL)		Ln CD31+/CD42a+ (counts/μL)			CIMT (μm)	
	No.	Adjusted β (S.E.)	*P*	Adjusted β (S.E.)	*P*	No	Adjusted β (S.E.)	*P*
Total	884						-0.027	<.001
Sex								
Female	536	-0.645 (0.103)	<0.001	-0.588 (0.132)	<0.001	533	-26.403 (4.011)	<0.001
Male	348	-0.718 (0.113)	<0.001	-0.595 (0.165)	<0.001	349	-31.512 (5.101)	<0.001
Age, years								
12-19	274	-0.535 (0.134)	<0.001	-0.515 (0.178)	0.004	274	-28.847 (5.545)	<0.001
20-30	610	-0.699 (0.094)	<0.001	-0.588 (0.132)	<0.001	608	-27.723 (3.911)	<0.001
BMI z score								
≤ -0.19	441	-0.573 (0.108)	<0.001	-0.603 (0.154)	<0.001	440	-26.071 (4.242)	<0.001
> -0.19	443	-0.774 (0.109)	<0.001	-0.615 (0.146)	<0.001	442	-30.676 (4.698)	<0.001
Smoking status								
Not active smokers	775	-0.616 (0.081)	<0.001	-0.559 (0.111)	<0.001	773	-27.151 (3.307)	<0.001
Active smoker	109	-0.993 (0.236)	<0.001	-0.907 (0.360)	0.013	109	-34.050 (10.647)	0.002
HOMA-IR								

≤ 0.90	441	-0.647 (0.113)	<0.001	-0.622 (0.168)	<0.001	441	-27.249 (4.082)	<0.001
> 0.90	443	-0.661 (0.105)	<0.001	-0.592 (0.136)	<0.001	441	-28.082 (4.781)	<0.001

Adjusted as in Model 2

Abbreviations: 4-t-OP: 4-tertiary-octylphenol; BMI z score: z score of body mass index; CIMT, carotid intima-media thickness; HOMA-IR: homeostasis model assessment of insulin resistance

Supplementary Table S2. Regression coefficients of covariates with CIMT and microparticles CD31+/CD42+ in structural equation model

	CIMT (um)				CD31+CD42+			
	Estimate	SE	T	*P*	Estimate	SE	T	*P*
4-tOP	-25.223	3.039	-8.300	<.001	-0.604	0.101	-5.969	<.001
CD31+/CD42+	3.685	0.993	3.371	<.001				
Gender	7.681	3.273	2.347	0.019	0.055	0.111	0.496	0.620
Age (year)	1.503	0.466	3.225	0.010	-.0.036	0.016	-2.288	0.022
Smoking	0.180	4.868	0.037	0.970	0.233	0.165	1.413	0.158
BMI z score	7.813	1.529	5.110	<.001	-0.007	0.052	-0.127	0.899
Systolic blood pressure (mmHg)	0.197	0.111	1.765	0.078	0.000	0.004	-0.044	0.965
LDL-C (mg/dL)	0.302	0.052	5.832	<.001	0.003	0.002	1.589	0.112
Ln-Triglyceride (mg/dL)	-6.599	3.412	-1.934	0.053	-0.498	0.115	-4.344	<.001
Ln-HOMA-IR	3.787	1.713	2.212	0.027	0.419	0.056	7.429	<.001
Model of fit								
GFI	1.000							
NFI	1.000							
RMS	0.001							

Supplementary Table S3. Regression coefficients of covariates with CIMT and microparticles CD31+/CD42− in structural equation model

	CIMT (um)				CD31+CD42−			
	Estimate	SE	T	*P*	Estimate	SE	T	*P*
4-tOP	-21.435	3.062	-7.000	<.001	-.662	.073	-9.068	<.001
CD31+/CD42−	9.090	1.352	6.721	<.001				
Gender	6.377	3.224	1.978	0.048	.166	.080	2.069	.039
Age (year)	1.804	0.461	3.912	0.001	-.048	.011	-4.200	<.001
Smoking	0.416	4.780	0.087	0.931	.069	.119	.576	.564
BMI z score	7.253	1.505	4.820	<.001	.059	.037	1.574	.116
Systolic blood pressure (mmHg)	0.188	0.110	1.714	0.087	.001	.003	.334	.738
LDL-C (mg/dL)	0.294	0.051	5.776	<.001	.002	.001	1.590	.112
Ln-Triglyceride (mg/dL)	-11.440	3.349	-3.416	<.001	.331	.083	4.001	<.001
Ln-HOMA-IR	3.369	1.659	2.031	0.042	.216	.041	5.306	<.001
Model of fit								
GFI	1.000							
NFI	1.000							
RMS	0.000							

References

1. Lin, C.Y.; Lin, L.Y.; Wen, T.W.; Lien, G.W.; Chien, K.L.; Hsu, S.H.; Liao, C.C.; Sung, F.C.; Chen, P.C.; Su, T.C. Association between levels of serum perfluorooctane sulfate and carotid artery intima-media thickness in adolescents and young adults. *International journal of cardiology* **2013**, *168*, 3309-3316, doi:10.1016/j.ijcard.2013.04.042.
2. WHO. Global Database on Child Growth and Malnutrition. Available online: http://www.who.int/nutgrowthdb/software/en/ (accessed on March 15).
3. Keskin, M.; Kurtoglu, S.; Kendirci, M.; Atabek, M.E.; Yazici, C. Homeostasis model assessment is more reliable than the fasting glucose/insulin ratio and quantitative insulin sensitivity check index for assessing insulin resistance among obese children and adolescents. *Pediatrics* **2005**, *115*, e500-503, doi:10.1542/peds.2004-1921.
4. Khoury, M.; Urbina, E.M. Hypertension in adolescents: diagnosis, treatment, and implications. *The Lancet. Child & adolescent health* **2021**, *5*, 357-366, doi:10.1016/s2352-4642(20)30344-8.
